# Accounting for multimorbidity can affect the estimation of the Burden of Disease: a comparison of approaches

**DOI:** 10.1186/s13690-016-0147-7

**Published:** 2016-08-22

**Authors:** Henk B. M. Hilderink, Marjanne H. D. Plasmans, Bianca E. P. Snijders, Hendriek C. Boshuizen, M. J. J. C. (René) Poos, Coen H. van Gool

**Affiliations:** 1 National Institute for Public Health and the Environment (RIVM), P.O. Box 1, 3720 BA Bilthoven, The Netherlands; 2 Wageningen University & Research Centre, Wageningen, The Netherlands

**Keywords:** Multimorbidity, Disease burden, Prevalence, Disability weights

## Abstract

**Background:**

Various Burden of Disease (BoD) studies do not account for multimorbidity in their BoD estimates. Ignoring multimorbidity can lead to inaccuracies in BoD estimations, particularly in ageing populations that include large proportions of persons with two or more health conditions. The objective of this study is to improve BoD estimates for the Netherlands by accounting for multimorbidity. For this purpose, we analyzed different methods for 1) estimating the prevalence of multimorbidity and 2) deriving Disability Weights (DWs) for multimorbidity by using existing data on single health conditions.

**Methods:**

We included 25 health conditions from the Dutch Burden of Disease study that have a high rate of prevalence and that make a large contribution to the total number of Years Lived with a Disability (YLD). First, we analyzed four methods for estimating the prevalence of multimorbid conditions (i.e. independent, independent age- and sex-specific, dependent, and dependent sex- and age-specific). Secondly, we analyzed three methods for calculating the Combined Disability Weights (CDWs) associated with multimorbid conditions (i.e. additive, multiplicative and maximum limit). A combination of these two approaches was used to recalculate the number of YLDs, which is a component of the Disability-Adjusted Life Years (DALY).

**Results:**

This study shows that the YLD estimates for 25 health conditions calculated using the multiplicative method for Combined Disability Weights are 5 % lower, and 14 % lower when using the maximum limit method, than when calculated using the additive method. Adjusting for sex- and age-specific dependent co-occurrence of health conditions reduces the number of YLDs by 10 % for the multiplicative method and by 26 % for the maximum limit method. The adjustment is higher for health conditions with a higher prevalence in old age, like heart failure (up to 43 %) and coronary heart diseases (up to 33 %). Health conditions with a high prevalence in middle age, such as anxiety disorders, have a moderate adjustment (up to 13 %).

**Conclusions:**

We conclude that BoD calculations that do not account for multimorbidity can result in an overestimation of the actual BoD. This may affect public health policy strategies that focus on single health conditions if the underlying cost-effectiveness analysis overestimates the intended effects. The methodology used in this study could be further refined to provide greater insight into co-occurrence and the possible consequences of multimorbid conditions in terms of disability for particular combinations of health conditions.

## Background

The Disability-Adjusted Life Year (DALY) is a widely used measure to quantify the burden of disease (BoD) in a population and to prioritize public health policy [1]. The DALY factors in premature mortality as expressed in Years of Life Lost (YLLs), and loss of quality of life due to suboptimal health status as expressed in Years Lived with a Disability (YLDs). The latter measure indicates the morbidity level, combining the occurrence of health conditions and their severity as represented by the Disability Weight (DW) [2, 3]. YLD calculations are based on a prevalence perspective, which is considered an adequate measurement of the level of disability experienced in a particular population at a particular moment in time [4]. The incidence perspective, on the other hand, combines the incidence of a particular event and its duration, and provides a measure of the loss of health connected with events in a given time period [Schroeder 2012]. Ignoring multimorbidity (i.e. co-occurrence of multiple health conditions within one person [5]), as has been done in various BoD studies so far, might result in overestimation of the number of YLDs and therefore overestimation of the overall disease burden [6, 7]. Also in Dutch studies, YLD calculations do not account for multimorbidity; for example, those included in the latest Public Health Status and Forecasts (PHSF) report [8]. This same publication, however, did recognize the importance of multimorbidity, reporting that in 2011, 1.9 million persons had two or more health conditions, representing 11 % of the Dutch population, with a projected increase to 3 million persons or 17 % by 2030. Multimorbidity occurs more often at older ages than at younger ages. Correcting for multimorbidity is therefore relevant to support policy intervention strategies, especially those aimed at an ageing population.

Accounting for multimorbidity in BoD studies requires not only estimates of the prevalence of multimorbid conditions, but also estimates of the severity of (two or more) health conditions [6]. Existing studies on the prevalence of multimorbid conditions are limited to combinations of two health conditions [9]. The Disability Weights associated with multimorbid conditions can be determined by means of a direct population sample, but this method is costly and time-consuming. Alternative approaches have been developed that derive the Disability Weights associated with multimorbid conditions from the underlying single health conditions. The three most frequently used methods are the additive, multiplicative and maximum limit methods [6, 10–13]. To date, there is a lack of studies providing insight into the possible effects on the disease burden of combining the approaches for determining the prevalence of multimorbid conditions and the associated Disability Weights.

The objective of this study is to apply different methods for estimating the prevalence of multimorbidity and estimating the Disability Weights associated with multimorbid conditions. In addition, we analyzed the effects that combining these methods would have on the number of YLDs. This analysis was performed using existing data concerning the prevalence of 25 selected health conditions in the Netherlands.

## Methods

**Fig. 1:**
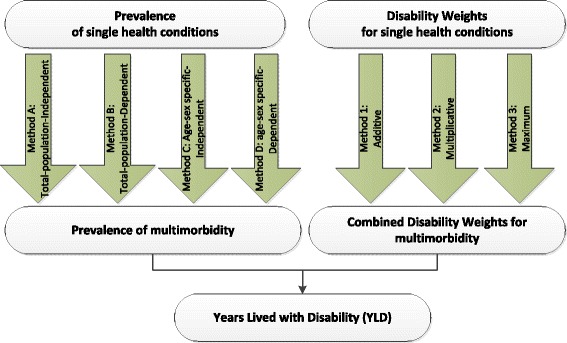
Various methodologies to account for multimorbidity prevalence and Disability Weights

In this context, multimorbidity is defined as the co-occurrence of multiple chronic or acute diseases and medical conditions within one person [5]. To take multimorbidity into account, various methods have been included in the analysis (see Fig. 1). These methods apply to the prevalence of multimorbidity (Fig. 1, left) as well as the Combined Disability Weights (CDWs) (Fig. 1, right). We included four methods for estimating the prevalence of multimorbidity and three methods for determining Combined Disability Weights, resulting in 12 variants for calculating the effect of multimorbidity on the BoD. As a reference for these variants, we used the approach applied in the PHSF report [7], which actually is one of the variants.

### Data and selection of health conditions

The data used to analyze multimorbidity consist of the data on the prevalence of health conditions and Disability Weights as published in the Dutch Public Health Status and Forecasts report (PHSF, [8]). The health conditions reported in the PHSF are considered the most relevant conditions in the Netherlands in terms of mortality, prevalence, cost, and impact on individuals and society [14]. Prevalence data for the health conditions have been derived from sources including GP registration databases and the Netherlands Cancer Registry, and are available by sex and for 5-year age cohorts, with persons over the age of 85 as the last age cohort. Disability weights were assigned to these health conditions based on the findings of the Dutch Disability Weights Study [15]. We have selected 25 health conditions for analysis, based on the following criteria: 1) high prevalence, 2) large contribution to the YLD component of the disease burden, and 3) variation in types (both mental and physical) (Table 1).

**Table 1:** Overview of the 25 health conditions included in the analysis: prevalence, Disability Weight (DW), Years Lived with a Disability (YLD), and Disability-Adjusted Life Years (DALY), 2011 [34]

	Prevalence	DW	YLD	DALY
Arthrosis	7.12 %	0.103	122,423	123,509
Anxiety disorders	5.77 %	0.187	180,220	180,272
Diabetes mellitus	5.00 %	0.198	165,150	194,312
Hearing disorders	4.86 %	0.109	88,344	88,344
Mood disorders	2.31 %	0.425	164,025	164,592
Neck and back pain	3.91 %	0.236	153,930	154,499
Coronary heart disease	3.62 %	0.288	174,090	282,834
Asthma	2.86 %	0.080	38,192	39,244
COPD	2.17 %	0.314	113,600	177,809
Contact eczema	1.94 %	0.070	22,720	22,720
Visual impairments	1.81 %	0.137	41,348	41,375
Cardiac arrhythmias	1.17 %	0.154	30,142	48,305
Stroke	1.11 %	0.609	113,147	191,320
Breast cancer	0.60 %	0.265	26,459	88,019
Heart failure	0.85 %	0.154	21,809	67,660
Intellectual disabilities	0.77 %	0.430	55,599	56,929
Personality disorders	0.60 %	0.273	27,438	27,438
Colon cancer	0.36 %	0.294	17,667	87,177
Prostate cancer	0.40 %	0.231	15,271	39,403
Dementia	0.48 %	0.678	54,744	112,130
Valve problems	0.46 %	0.118	9091	28,346
Skin cancer	0.24 %	0.070	2768	19,446
Lung cancer	0.12 %	0.285	5845	169,120
Parkinson’s disease	0.19 %	0.497	15,401	25,657
Non-Hodgkin’s lymphoma	0.13 %	0.233	4910	20,749

### Different approaches to estimating the prevalence of multimorbidity

Various methods are used to calculate the prevalence of combinations of health conditions, based on existing data on the prevalence of single health conditions (Fig. 1):

- In Method A and C, health conditions are considered independently, as assumed in the GBD studies for 2010 and 2013 [3, 16]. This means that the occurrence of one health condition is assumed to have no effect on the occurrence of another health condition. In Method A, the calculations are performed for the entire population (non-age-specific and non-sex-specific). In Method C, calculations are stratified by gender and by 5-year age cohort.
- In Method B and D, the occurrence of health conditions is assumed to be interdependent. This is taken into account in the calculations by applying a dependence correction factor. In this study, dependence is only applied to combinations of two health conditions, due to a lack of insight into and data about combinations of more than two conditions. Combinations of more than two health conditions are regarded as occurring independently, as in Method A and C. Method B is applied to the entire population (non-age-specific and non-sex-specific). In Method D, the calculations are stratified by gender and by 5-year age cohort.

Method A and C represent the most straightforward approach, and assume that health conditions occur independently. Suppose we have *n* health conditions and the probability of having health condition *i* is *p*_*i*_ for *i* in 1 … *n*. The probability of having both health conditions *i* and *j* is:

1 $$ {p}_{ij,\  indep}={p}_i*{p}_j $$

The probability of having only two health conditions *i* and *j*, and no other health condition, is:

2 $$ {p}_{ij,\  indep}\hbox{'}=\frac{p_i*{p}_j*\left(1-{p}_1\right)*\dots *\left(1-{p}_n\right)}{\left(1-{p}_i\right)*\left(1-{p}_j\right)} $$

For 25 health conditions, there are more than 33 million possible combinations. Calculating the prevalence of these combinations requires a great deal of computing capacity. However, when health conditions are assumed to occur independently, the probability of having more than five health conditions is very small (no more than 1.5 in a million). We therefore limited our analyses to combinations of no more than five health conditions out of a total of 25 health conditions, which resulted in 68,405 unique combinations.

This approach disregards two important issues. Firstly, different health conditions may have shared risk factors (e.g. smoking not only increases the risk of a stroke but also of developing COPD). Secondly, some health conditions may increase the risk of getting another health condition, e.g. diabetes mellitus and cardiovascular diseases. As a result, some combinations of health conditions occur more frequently than might be expected if independence is assumed. Odds ratios (i.e. the ratio of the odds of the observed prevalence of two health conditions compared to the prevalence when independence is assumed) can be used to calculate the prevalence corrected for dependence. Van Oostrom et al. [17] published the odds ratios for nine health conditions (i.e. the ratio between observed and independent prevalence) in an older population. Only eight of these health conditions appear in our list of 25 health conditions. Since odds ratios for combinations of the 25 health conditions are not available, we used the median of the odds ratios reported by Van Oostrom et al. [17] (i.e. an odds ratio of 1.3). These odds ratios are only applied to combinations of two health conditions, since no data are currently available on odds ratios for combinations of more than two health conditions. This adjustment for interdependence between the prevalence of two health conditions is included in Method B and D.

The ratio *OR*_*ij*_ between the odds of the real prevalence *p*_*ij*,*dep*_ assuming interdependence between two health conditions *i* and *j*, and the odds of the prevalence assuming independence *p*_*ij*, *indep*_ between those two conditions may be calculated as follows:

3 $$ O{R}_{ij} = \frac{p_{ij,\ dep}}{\left(1-{p}_{ij,dep}\right)}/\frac{p_{ij,\  indep}}{\left(1-{p}_{ij,\  indep}\right)} $$

This formula can be rewritten as:

4 $$ {p}_{ij,\ dep}=O{R}_{ij}*\left(\frac{p_{ij, indep}}{1-{p}_{ij,\  indep}}\right)/\left(1 + O{R}_{ij}*\left(\frac{p_{ij,\  indep}}{1-{p}_{ij,\  indep}}\right)\right) $$

When odds ratios are used, *p*_*ij*, *dep*_ is always between 0 and 1. When the odds ratios are known, *p*_*ij*, *dep*_ can be calculated for each combination of two health conditions. In all methods, these probabilities replace the probabilities of the occurrence of combinations of two health conditions based on independence. In order to keep the total probability of health condition *i* and *j* the same, the probability that people only have health condition *i* (*p*_*i*_ ') or *j* (*p*_*j*_ ') is adjusted as follows:

5 $$ {p}_{i,dep}^{\hbox{'}}={p}_i\hbox{'}-\left({p}_{ij,\ dep} - {p}_{ij,\  indep}\right) $$

and

6 $$ {p}_{j,dep}^{\hbox{'}}={p}_j\hbox{'}-\left({p}_{ij,dep} - {p}_{ij, indep}\right) $$

The probability *p*_0_ of having none of the *n* health conditions is adjusted as follows:

7 $$ {p}_{0,dep}={p}_0+{\displaystyle \sum_{i=1}^n}{\displaystyle \sum_{j=1,j\ne i}^n}\left({p}_{ij,\ dep} - {p}_{ij,\  indep}\right) $$

Calculating the prevalence by gender and age may result in a lower prevalence for some combinations of health conditions compared to calculations for the population as a whole. For example, asthma is more prevalent among young people, and will therefore not occur as often in combination with old-age-related health conditions such as dementia. On the other hand, dementia will occur more often in combination with other chronic health conditions like arthrosis because of the age-related nature of these conditions.

### Different approaches to Combined Disability Weights (CDWs)

To adjust the Disability Weights for multimorbidity, we applied three approaches: the additive approach (Method 1), the multiplicative approach (Method 2) – as assumed in the GBD studies for both 2010 and 2013 [3, 16] – and the maximum limit approach (Method 3). These three methods exclude the possibility that a combined multimorbidity effect in terms of disability can be higher than the sum of the underlying disabilities. At the individual level, there may be combinations that could result in so-called over-additivity. We have assumed that over-additivity is less relevant at population level, and have therefore not included it in our analysis.

Method 1 (Fig. 1) represents an additive model in which the resulting impact of combined health conditions is defined as the sum of the impacts of the individual health conditions. It is assumed that the impact of each health condition is the same, regardless of the presence of other conditions. The Combined Disability Weight of health conditions *i* and *j* is therefore calculated as follows:

8 $$ D{W}_{ij}=D{W}_i+D{W}_j $$

where *DW*_*i*_ is the Disability Weight of health condition *i*, and *DW*_*j*_ is the Disability Weight of health condition *j*.

Method 2 represents a multiplicative model in which each health condition proportionally contributes to the Combined Disability Weight. The Combined Disability Weight of health conditions *i* and *j* is therefore calculated as follows:

9 $$ D{W}_{ij}=1-\left(1-D{W}_i\right)*\left(1-D{W}_j\right) $$

In Method 3, the maximum limit approach ignores co-existing health conditions and assumes that the most serious condition “trumps” the others. The Combined Disability Weight of health conditions *i* and *j* is therefore calculated as follows:

10 $$ D{W}_{ij}= \max \left(D{W}_i,\ D{W}_j\right) $$

### Years Lived with a Disability (YLD)

The overall objective is to estimate the health loss that is associated with a specific health condition, as well as the overall health loss from all health conditions occurring in a population. The number of Years Lived with a Disability (YLD) for health condition *i* is considered from a prevalence perspective [4], and may be calculated as follows:

11 $$ YL{D}_i={p}_i*D{W}_i $$

And for the total population:

12 $$ YL{D}_{total\  population}={\displaystyle \sum_{i = 1}^n}{p}_i*D{W}_i $$

where *p*_*i*_is the prevalence of health condition *i*, *DW*_*i*_ is the corresponding Disability Weight, and *n* is the total number of health conditions occurring in a population. This calculation implicitly assumes that when a person has more than one health condition, the disabilities associated with these health conditions may be added up (Eq. 12). This assumption most likely results in an overestimation of the total number of YLDs in the population.

In order to determine how much of the disability can be attributed to a specific health condition, the fraction of the Disability Weight (DWF) that is attributable to a specific health condition is calculated [18]. If a person has *k* different health conditions with corresponding Disability Weights *DW*_1_ … *DW*_*k*_, the DWF attributable to health condition *i* may be calculated as follows*:*

13 $$ DW{F}_i=\frac{D{W}_i}{D{W}_1+\dots +D{W}_k} $$

The attributable Disability Weight *DWA*_*i*_ caused by health condition *i* in this person may be calculated as follows:

14 $$ DW{A}_i=DW{F}_i*D{W}_{1\dots k} $$

where *DW*_1 … *k*_ is the Combined Disability Weight of health conditions 1 … *k*

If these formulas are applied to the three different methods for calculating Combined Disability Weights, the results are as follows:

15 $$ \mathrm{Method}\ 1:DW{A}_i=DW{F}_i*D{W}_{1\dots k}=\frac{D{W}_i}{D{W}_1+\dots +D{W}_k}*\left(D{W}_1+\dots +D{W}_k\right)=D{W}_i $$

16 $$ \mathrm{Method}\ 2:DW{A}_i=DW{F}_i*D{W}_{1\dots k}=\frac{D{W}_i}{D{W}_1+\dots +D{W}_k}*\left(1-\left(1-D{W}_1\right)*\dots *\left(1-D{W}_k\right)\right) $$

17 $$ \mathrm{Method}\ 3:DW{A}_i=DW{F}_i*D{W}_{1\dots k}=\frac{D{W}_i}{D{W}_1+\dots +D{W}_k}* \max \left(D{W}_1,\dots,\ D{W}_k\right) $$

Suppose *t* people in a population have health condition *i.* The YLDs attributable to health condition *i* in the entire population may be calculated as follows:

18 $$ YL{D}_i={\displaystyle \sum_{p=1}^t}DW{A}_{ip} $$

where *DWA*_*ip*_ is the attributable Disability Weight caused by health condition *i* in a person *p.*

The analyses were performed using R version 3.1.0. The R-scripts are available upon request.

## Results

### Prevalence of multimorbidity

**Fig. 2:**
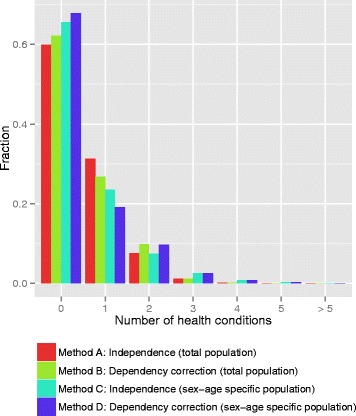
Prevalence of average number of health conditions in a person (determined using the four methods for calculating the prevalence of combinations of health conditions)

Figure 2 shows that, when independence is assumed between health conditions in the overall study population (i.e. applying Method A), almost 60 % of the population does not have any health condition, about 30 % has one of the selected health conditions, 8 % has two health conditions, and 3 % has three or more health conditions. When independence between health conditions is assumed in age- and sex-specific analyses (i.e. Method C), the results show that 35 % of the population has at least one health condition (Fig. 2). When Method B is applied and interdependence between different health conditions is assumed, the prevalence is slightly lower for single health conditions and slightly higher for double health conditions compared to the results produced by Method A.

**Fig. 3:**
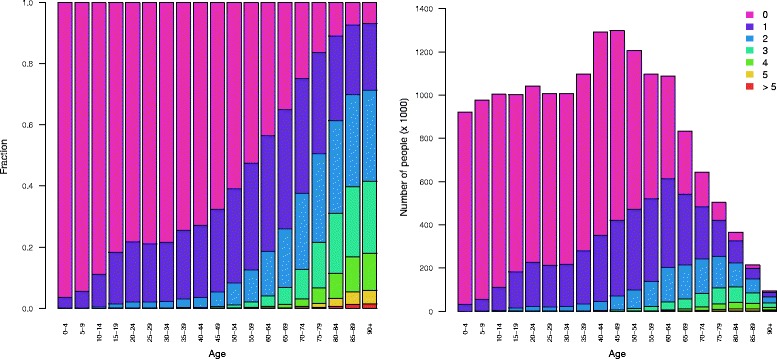
Relative (*left*) and absolute (*right*) prevalence of average number of health conditions in a person by age, determined using Method C

Figure 3 shows the relative (left) and absolute (right) age- and sex-specific prevalence of the average number of health conditions in a person, determined in accordance with Method C. A clustering of health conditions occurs at higher ages (75 years and over). The proportion of the population without any health conditions is 10 % in the 75+ age cohort, and around 60 % has two or more health conditions. This age-specific pattern is similar for men and women in relative terms, although women outnumber men at higher ages in absolute terms, resulting in a higher contribution by women to the health condition burden (results included in Appendix).

**Fig. 4:**
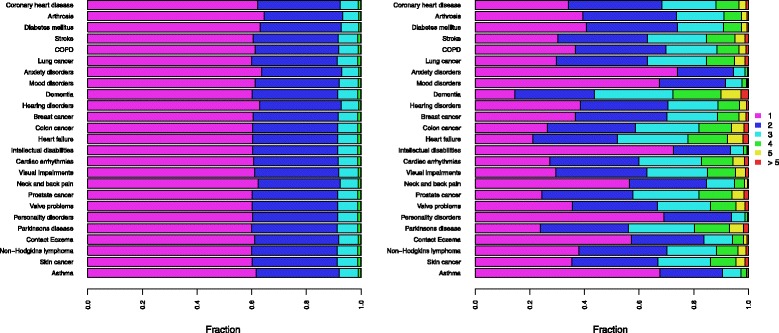
Total-population (Method A, *left*) and sex- and age-specific (Method C, *right*) calculated prevalence of average number of health conditions in a person, by health condition

Looking at specific health conditions, there is a distinct difference between total-population methods (Methods A and C) and sex- and age-specific methods (Methods B and D). Figure 4 shows the differences between Method A and Method C. Although the overall prevalence of health conditions displays a relatively wide range of variation (Table 1), the probability of occurrence of a single health condition either by itself or in combination with one or more of the other 24 health conditions shows little variation in Method A (Fig. 4, left). This can be explained by looking at the probability of not having any other health condition, which has a strong commonality for different health conditions. In Method C and D, the underlying age-specific variation in prevalence results in greater overall variation. Applying Method C (Fig. 4, right) shows that the vast majority of dementia cases (almost 90 %) occur in combination with another health condition, due to a clustering of chronic health conditions at higher ages. In contrast, anxiety disorders may be regarded as a health condition typical of middle age and co-occur with another health condition in only 30 % of cases, assuming independent occurrence.

### Three different methods for calculating Combined Disability Weights

**Fig. 5:**
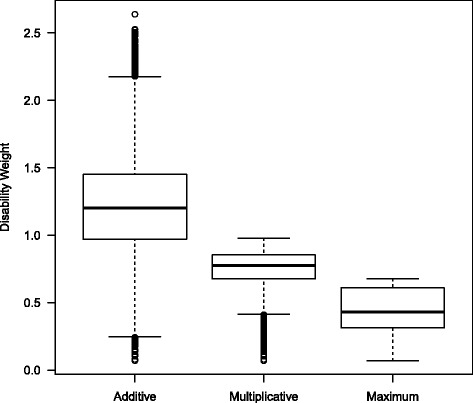
Box plot of the distribution of Disability Weights for the three methods for calculating Combined Disability Weights

The Disability Weights for the 25 selected health conditions range from 0.070 to 0.678 (Table 1). Figure 5 shows the distribution of Disability Weights when three different calculation methods are applied, for all possible combinations of health conditions. These combinations concern the simultaneous occurrence of one to five health conditions. When looking at a combination of five health conditions, the Combined Disability Weights range from 0.432 to 2.639 when the additive method is applied. By definition, the Disability Weight should not exceed 1 (which corresponds with death), although this may occur when the additive method is applied. When the multiplicative method is applied, the Disability Weights are lower and have a smaller range (0.364 - 0.979). Application of the maximum limit method results in even lower Disability Weights (0.109 - 0.678). In general, the maximum limit method (Method 3) results in the largest downward adjustment of the Combined Disability Weight (CDW) compared to the additive method. When the multiplicative method is applied, the resulting CDWs are higher than when Method 3 is applied, but lower than when Method 1 is applied (see Fig. 5).

### Impact of different methods for calculating prevalence and Disability Weight on YLD estimates

**Fig. 6:**
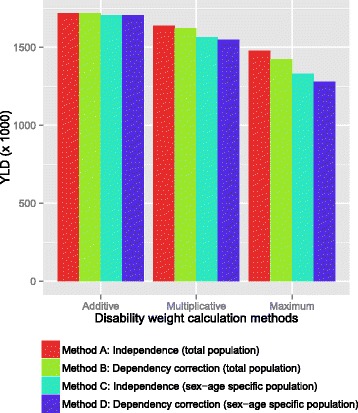
YLD estimates determined using the methods for estimating prevalence and Disability Weights

Combining the methods for calculating the prevalence of multimorbidity and the methods for calculating the Disability Weights of multimorbid conditions results in twelve disease burden outcomes for the selected 25 conditions, expressed in YLDs (Fig. 6). The YLDs calculated using Method A (no sex or age specificity) in combination with Method 1 (additive method) actually correspond to not taking multimorbidity into account, and amount to a total of 1.72 million years lived with disability for the 25 selected health conditions. This combination serves as a reference for the other eleven combinations. Calculating YLDs based on the assumption of independent prevalence (Method A) in combination with the multiplicative approach (Method 2) for combining Disability Weights – which corresponds to the perspective of the GBD studies [3, 16] – yields 1.64 million years lived with a disability. This corresponds to a downward adjustment of 4.6 % compared to not taking multimorbidity into account. Calculating the sex- and age-specific prevalence assuming interdependence (Method D) in combination with the maximum limit method (Method 3) results in the largest downward YLD adjustment, by 26 % to a level of 1.28 million years lived with a disability. The YLD adjustment when the multiplicative method (Method 2) is used, amounts to 10 % for sex- and age-specific prevalence (Method D).

**Fig. 7:**
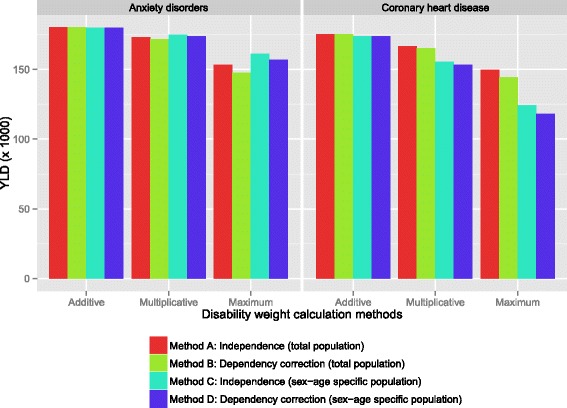
YLD estimates for anxiety disorders and coronary heart disease, determined using the different methods

Looking at specific health conditions, anxiety disorders are associated with a moderate adjustment of the related-disease burden (up to 13 %, Fig. 7), because of a relative high prevalence in middle age (25–45 years). The health conditions that are associated with a high adjustment, such as coronary heart disease (up to 33 %) and heart failure (up to 43 %), all show a strong increase in prevalence with age.

The Appendix includes a table that provides the results for all twelve methods, for all 25 selected health conditions.

## Discussion

A key focus area in multimorbidity research is the development of tools to explore multimorbidity and its impact on, for example, burden of disease, disability and quality of life [19]. Various Burden of Disease (BoD) studies do not account for multimorbidity in their BoD estimates. In this study, we applied four different methods to estimate the prevalence of multimorbidity, and three different methods to calculate the Disability Weights associated with multimorbid conditions. This resulted in twelve different calculations of the number of Years Lived with a Disability (YLDs), in order to analyze the impact of multimorbidity on BoD estimates in the Netherlands. We found that multimorbidity adjustments can have a substantial impact on YLD estimates for the 25 health conditions included in our analysis. When a multiplicative method is applied to determine Combined Disability Weights, the YLDs are 5 % lower than when the additive method is used, and 14 % lower than when the maximum limit method is used. Adjusting for the sex- and age-specific dependent co-occurrence of health conditions reduces YLD estimates by 10 % when the multiplicative method is used, and by 26 % when the maximum limit method is used.

Considering the four different methods for estimating the prevalence of multimorbidity, we found the highest level of clustering of health conditions in the sex- and age-specific calculation (Methods C and D), i.e. a relatively high occurrence of multimorbidity. Some studies have estimated the overall prevalence of multimorbidity in both the general population and in primary care settings, leading to marked variations among studies with respect to both methodology and findings [20–22]. Based on an analysis of registration data for 29 health conditions, Van Oostrom et al. [17] found that 17 % of primary care patients in the Netherlands are disease-free and 59 % have two or more health conditions [17].

When looking at specific clusters of health conditions, our approach to estimating the prevalence of multimorbidity resulted in a stronger correlation between health conditions that progress with age, such as dementia and cardiovascular diseases. We did not adjust for possible higher interdependence between specific combinations of health conditions. In a systematic review of data on older adults with multiple chronic diseases, it was found that the combinations with the highest prevalence rates included hypertension, coronary artery disease and diabetes mellitus [20]. A German study found three multimorbidity patterns through both factor analysis and network analysis: 1) cardiovascular/metabolic disorders, 2) anxiety/depression/somatoform disorders and pain, and 3) neuropsychiatric disorders [23, 24]. In general, there is limited insight into the prevalence of specific disease clusters, especially for combinations of more than two health conditions. Due to this lack of data, the value of the dependence correction factor used in our analyses (1.3) is based on the median reported in the literature. This factor may be lower or higher for certain combinations (e.g. cardiovascular diseases show odds ratios of 5.9 [9]). Sensitivity analyses show a negative linear correlation between the dependence correction factor and the YLD estimates (see Appendix). In our analysis, dependence between health conditions is limited to combinations of two health conditions, since adequate information about the occurrence of more than two health conditions is lacking. This implies that the prevalence of multimorbidity adjusted for dependence between health conditions is underestimated, and that a larger downward YLD adjustment should therefore be applied.

In addition to methods for estimating the prevalence of multimorbidity, we explored three different methods for determining the Combined Disability Weight of multimorbidity. The simultaneous occurrence of multiple health conditions may have less impact on a person’s health than might be expected based on the sum of the impacts of the individual health conditions. However, there is no golden standard for estimating Combined Disability Weights. There are specific findings about applying these methods, and an alternative non-parametric method has even been developed. This so-called adjusted decrement estimator method is a variation on the maximum limit method ([11, 13]). However, many studies [13, 25, 26] use utility measures such as EQ-5D scores or Health Utilities Index Mark 3 (HUI3) instead of Disability Weights, resulting in profound differences. Haagsma et al. [12] compared three comorbidity approaches in patients with temporary injury consequences as well as comorbid chronic conditions with non-trivial health impacts. They found that the Disability Weight of injury patients increases proportionally to the number of comorbid health conditions. The Disability Weights in the study by Haagsma et al. were based on EQ-5D scores [12], while in our study the Disability Weights were derived from the Dutch Disability Weights Study [15]. The most effective method in each case depends to a large extent on the available data, and most studies conclude that further research is required to validate the results found [11, 25, 27–30].

This study focuses on accounting for multimorbidity to produce more accurate YLD estimates. However, one could argue that a similar approach may be applied to YLL estimates. Accounting for “multiple causes of death” – i.e. accounting not only for primary causes of death but also for secondary or even tertiary causes – could alter the allocation of YLLs to specific causes of death [31, 32]. Research on this is still in its infancy, however.

## Conclusions

Burden of Disease (BoD) calculations that do not account for multimorbidity can result in an overestimation of the real BoD. This may affect public health policy strategies that focus on single health conditions [33]. For instance, cost-effectiveness analyses might overestimate intended effects when focusing on one particular health condition without accounting for multimorbidity. Furthermore, applying the independent prevalence method (Method A) in combination with the multiplicative approach (Method 2) for combining Disability Weights is a preferred approach to account for multimorbidity. This approach to YLD estimates is relatively simple, and may serve as the preferred approach until more insight has been gained into the dependent co-occurrence of health conditions and the consequences of multimorbid conditions in terms of Disability Weight.

## Abbreviations

BoD, Burden of Disease; CDW, combined disability weight; COPD, chronic obstructive pulmonary disease; DALY, disability-adjusted life years; DW, disability weight; DWA, disability weight attributable to a particular disease; OR, odds ratio; YLD, years lived with disability; YLL, years of life lost

## Figures and Tables

**Fig. 8:**
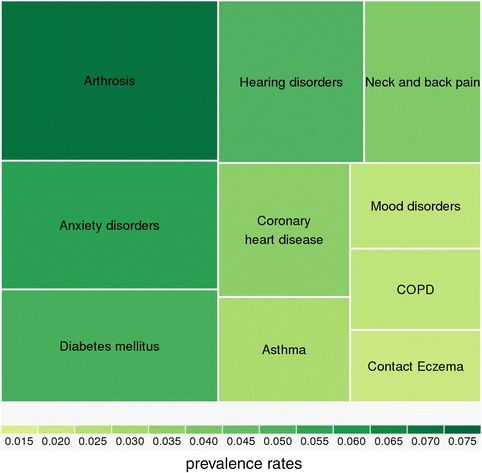
Top 10 of health conditions with the highest prevalence rates

**Fig. 9:**
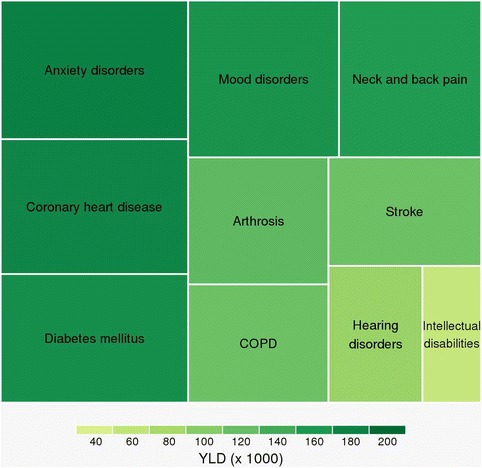
Top 10 of health conditions with the highest YLD

**Fig. 10:**
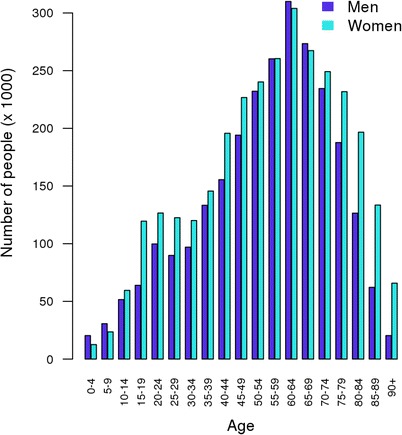
Number of people with more than one health condition by sex and age (Method C)

**Fig. 11:**
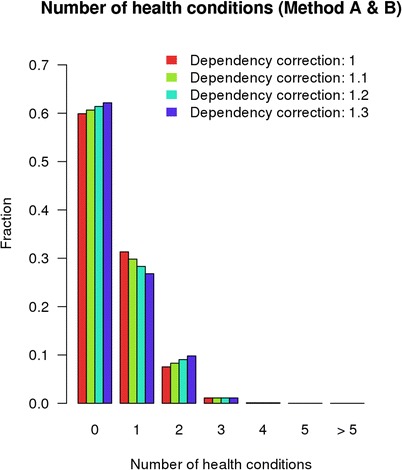
Prevalence of average number of health conditions in a person for different dependency correction factors

**Fig. 12:**
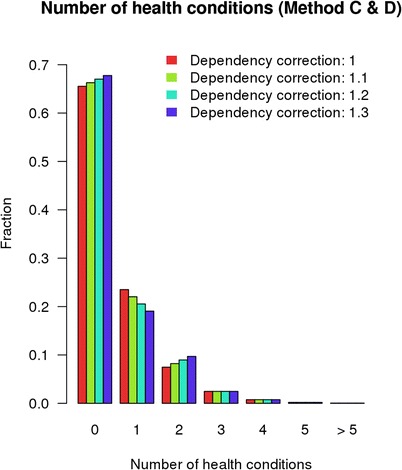
Prevalence of average number of health conditions in a person for different dependency correction factors

**Fig. 13:**
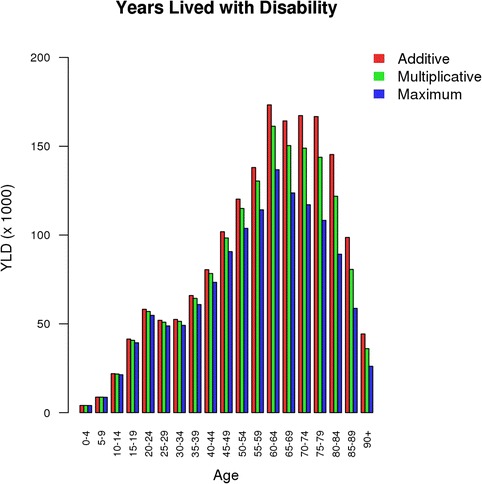
Years Lived with Disability (YLDs) by age (Method C)

**Fig. 14:**
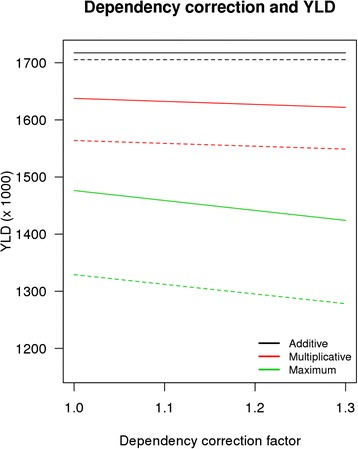
Dependency correction factor and YLD (*dashed lines* represent sex- age-specific method)

## Appendix

**Table 2:** Results in YLDs for all twelve methods, for all 25 health conditions

	Independent total-population additive	Independent total-population multiplicative	Independent total-population maximum limit	Dependent total-population additive	Dependent total-population multiplicative	Dependent total-population maximum limit	Independent, sex-age additive	Independent, sex-age multiplicative	Independent, Sex-age maximum limit	Dependent, sex-age additive	Dependent, sex-age multiplicative	Dependent, sex-age maximum limit
Anxiety disorders	180,000	173,000	153,000	180,000	172,000	147,000	180,000	175,000	161,000	180,000	174,000	157,000
Coronary heart disease	175,000	167,000	150,000	175,000	165,000	144,000	174,000	155,000	124,000	174,000	153,000	118,000
Diabetes mellitus	167,000	160,000	142,000	167,000	159,000	136,000	166,000	152,000	122,000	166,000	151,000	116,000
Mood disorders	164,000	155,000	143,000	164,000	153,000	139,000	164,000	156,000	146,000	164,000	154,000	143,000
Neck and back pain	155,000	148,000	131,000	155,000	146,000	126,000	154,000	145,000	125,000	154,000	143,000	120,000
Arthrosis	124,000	120,000	107,000	124,000	119,000	104,000	123,000	114,000	92,000	123,000	113,000	88,000
COPD	115,000	109,000	98,000	115,000	108,000	95,000	114,000	102,000	83,000	114,000	101,000	79,000
Stroke	115,000	107,000	103,000	115,000	106,000	100,000	113,000	97,000	88,000	113,000	95,000	85,000
Hearing disorders	89,000	86,000	76,000	89,000	86,000	74,000	88,000	82,000	65,000	88,000	81,000	62,000
Intellectual disabilities	56,000	52,000	48,000	56,000	52,000	47,000	56,000	53,000	50,000	56,000	53,000	49,000
Dementia	54,000	51,000	49,000	54,000	50,000	48,000	53,000	42,000	38,000	53,000	42,000	37,000
Breast cancer	47,000	45,000	40,000	47,000	45,000	38,000	47,000	42,000	34,000	47,000	42,000	32,000
Visual impairments	42,000	40,000	35,000	42,000	40,000	34,000	41,000	37,000	28,000	41,000	37,000	26,000
Asthma	38,000	37,000	33,000	38,000	37,000	32,000	38,000	37,000	34,000	38,000	37,000	33,000
Cardiac arrhythmias	30,000	29,000	25,000	30,000	29,000	24,000	30,000	27,000	20,000	30,000	27,000	19,000
Colon cancer	30,000	28,000	25,000	30,000	28,000	24,000	29,000	26,000	19,000	29,000	25,000	18,000
Personality disorders	27,000	26,000	23,000	27,000	26,000	22,000	27,000	26,000	24,000	27,000	26,000	23,000
Contact Eczema	23,000	22,000	20,000	23,000	22,000	19,000	23,000	22,000	19,000	23,000	22,000	19,000
Heart failure	20,000	20,000	17,000	20,000	19,000	16,000	20,000	18,000	12,000	20,000	17,000	12,000
Prostate cancer	19,000	18,000	16,000	19,000	18,000	15,000	19,000	16,000	12,000	19,000	16,000	11,000
Parkinsons disease	15,000	14,000	13,000	15,000	13,000	12,000	14,000	12,000	10,000	14,000	12,000	10,000
Lung cancer	12,000	12,000	10,000	12,000	12,000	10,000	12,000	11,000	8,000	12,000	11,000	8,000
Valve problems	9,000	9,000	8,000	9,000	9,000	7,000	9,000	8,000	6,000	9,000	8,000	6,000
Non-Hodgkins lymphoma	5,000	5,000	4,000	5,000	5,000	4,000	5,000	4,000	3,000	5,000	4,000	3,000
Skin cancer	5,000	5,000	4,000	5,000	5,000	4,000	5,000	4,000	4,000	5,000	4,000	3,000
Total (25 health conditions)	1,717,000	1,638,000	1,476,000	1,717,000	1,622,000	1,424,000	1,705,000	1,564,000	1,329,000	1,705,000	1,549,000	1,278,000
